# Understanding the burden of smokeless tobacco use in rural India: patterns, motivations, and public health implications

**DOI:** 10.3389/fpubh.2026.1850268

**Published:** 2026-10-08

**Authors:** Sweta Kale Pisulkar, Sonali Choudhari, Shruti Deshmukh

**Affiliations:** 1 Department of Prosthodontics and Crown and Bridge, Sharad Pawar Dental College and Hospital, Wardha, India; 2 Department of Community Medicine, Jawaharlal Nehru Medical College, Datta Meghe Institute of Higher Education & Research, Wardha, India

**Keywords:** health risk awareness, LSAI, public health, rural India, smokeless tobacco

## Abstract

**Background:**

The use of smokeless tobacco (SLT) is a major public health problem in India, particularly in rural areas, where it is accepted and easily accessible due to a lack of awareness. Despite government initiatives to control tobacco use, SLT is a cause of serious illness and death.

**Aim:**

This study aims to evaluate the prevalence, patterns, and socio-demographic determinants of smokeless tobacco use in rural India using data from Longitudinal Survey on Addictive Influences (LSAI).

**Materials and methods:**

A cross-sectional analytical study was carried out using secondary data from the LSAI dataset. Rural participants aged ≥18 years were included. Age, gender, education, occupation, socioeconomic status, and the type and frequency of SLT usage were among the variables examined. Descriptive statistics were used to find prevalence and patterns, whereas chi-square test and logistic regression analysis were used to find the determinants associated with SLT use.

**Results:**

Among the rural population, the use of smokeless tobacco was found to be highly prevalent, with men using it much more frequently than women. The two most frequently consumed forms of SLT were Gutkha and Khaini. There were statistically significant associations between the use of SLT and age, gender, education, and socioeconomic status (*p* < 0.05). Individuals with lower levels of education and those who were manual laborers had a higher chance of using SLT. Early initiation and daily use patterns were common, indicating strong dependence.

**Conclusion:**

In rural India, the use of smokeless tobacco is still prevalent, and the main contributing factors are the inequalities in socioeconomic status and education. The use of SLT and the health risks associated with it should be decreased by public health interventions, which should include education and stricter enforcement of tobacco control laws.

## 1 Introduction

Smokeless tobacco (SLT) usage is a serious public health issue in India, particularly high usage rates have been observed in rural areas of Indian population. It is one of the major causes of death and disease in India and accounts for nearly 1.35 million deaths every year. India is also the second-largest consumer and producer of tobacco. Nearly 267 million adults (15 years and above) in India (29% of all adults) are users of tobacco, according to the Global Adult Tobacco Survey India, 2016–17.

Unlike other forms of tobacco, SLT does not involve burning and comprises a range of different products, which include gutkha (a mixture of areca nut, slaked lime, and flavored tobacco), khaini (sun-dried tobacco with lime), zarda (processed flavored tobacco), and betel quid (a mixture of betel leaves, areca nut, and tobacco). These products are usually widely available, affordable, and have strong link with local traditions and practices, which makes them popular in rural areas (1).

Acceptance by culture, a lack of understanding of health risks, and tradition of use are all considerations that contribute to the long-standing tradition of SLT consumption in these communities. In some rural areas, the use of SLT is not only a matter of personal choice but also a social one. It is often served at social gatherings, religious events, and even during agricultural breaks, thus further ingraining its acceptance. Women, youth and even children are sometimes exposed to SLT from a young age, often as a family tradition or to relieve toothaches, hunger, or stress.

The Global Adult Tobacco Survey (GATS) is a standardized assessment carried out to observe tobacco usage among adults and to monitor essential tobacco control metrics. In India, GATS was performed during the years 2009–2010 and 2016–17, encompassing all states and Union Territories. The objective of the survey is to improve countries' abilities to formulate, execute, and assess tobacco control initiatives, offering data that can be compared internationally (2).

According to the GATS survey, the number of SLT consumers in India is more than 200 million, which is greater than 70% of the total number of SLT consumers around the world. This effect is mainly observed in the rural areas due to a variety of reasons, such as poverty, illiteracy, lack of proper health facilities, and lack of awareness among the general public. In spite of the strict laws and regulations regarding tobacco consumption in the country, the consumption of SLT is thriving in these areas, which are still not regulated. In summary, the health effects of SLT consumption are serious and alarming. One of the most dreadful health effects of SLT consumption is the development of oral cancer, which is the highest in the world (2).

According to scientific evidence, a strong association has been established between the consumption of SLT and the development of oral, pharyngeal, and esophageal cancers. In addition to the risk of oral cancer, the consumption of SLT is also known to result in a variety of chronic and debilitating health conditions, such as cardiovascular diseases, hypertension, myocardial infarction, stroke, periodontal diseases, submucous fibrosis, and reproductive health problems. Additionally, the addictive potential of nicotine which is found in SLT products can cause physical dependence and withdrawal symptoms, making it difficult to quit (3).

The psychosocial consequences of SLT product usage are also alarming. Tobacco addiction may cause productivity loss, economic consequences (especially among the poor), and increased healthcare expenses. Additionally, tobacco addiction may cause the continuation of poverty and poor health conditions, especially among the rural population where more than one member of the family may be a regular addict (4).

SLT products are easily available and promoted in the market despite the government's efforts through the Cigarettes and Other Tobacco Products Act (COTPA), pictorial warnings on the products, prohibition on public advertisements, and school education programs (5). However, the consequences of these efforts are further complicated by the lack of enforcement, the tobacco black market, surrogate advertisements, and the availability of local unregulated tobacco products. In addition, tobacco products are also considered safer than smoking and therefore do not require quitting (5, 6).

In order to properly grapple with the issue of SLT use, public health interventions must extend beyond the common practices of creating public awareness. There is an urgent need to develop culture-specific interventions to deal with the deeply ingrained social values that govern the use of SLT. This will include educationally targeted interventions, community mobilization through key opinion leaders, tobacco cessation service delivery through primary healthcare, and providing economic opportunities to people who are engaged in the cultivation and sale of SLT (7, 8).

In this regard, the Longitudinal Survey on Addictive Influences (LSAI) (15) presents a unique opportunity to investigate the dynamics of SLT use in-depth (7). By studying individuals and communities over time at different points, LSAI enables researchers to identify patterns of SLT behavior and the factors that influence continued or stopped use, and the impact of socio-economic and environmental changes on consumption patterns. Such longitudinal data is critical in differentiating between causality and association, enabling more effective formulation of policy (9).

The objectives of this study is to examine the pattern and determinants of smokeless tobacco use in rural India using LSAI data. This study analyzed the data gathered over time to identify the complex interplay of culture, behavioral, and socio-economic factors affecting the maintenance of SLT use (10, 11). This study seeks to contribute to the development of effective tobacco cessation strategies for people consuming tobacco that take into account the special circumstances and challenges of rural India in the global fight against tobacco use and its consequent morbidity and mortality.

## 2 Material and methodology

The present study was conducted to identify the patterns and aspects associated with the consumption of smokeless tobacco (SLT) in the lives of rural populations in Maharashtra. This section deals with the framework that was adopted for the overall methodology that was to be used in the course of the study.

### 2.1 Study design

This is a cross-sectional study, and the study utilizes the data collected during the baseline of the Longitudinal Survey on Addictive Influences (LSAI). LSAI is a large-scale survey that systematically collects data on addictive influences such as the use of tobacco, alcohol, and other substances. In the current study, the emphasis was given to the cross-sectional analysis of the data collected on the use of SLT among adults residing in rural areas of Maharashtra.

The cross-sectional study was considered appropriate for the study because it can be utilized for the estimation of the use of tobacco and the identification of sociodemographic correlates and behavioral patterns of the use of SLT.

### 2.2 Study setting

The research was conducted in the rural areas of the chosen districts (i.e., Wardha, Yavatmal, Chandrapur)of Maharashtra, which is a state with a higher prevalence of the use of SLT. The districts were chosen on the basis of the data collected from the previous health surveys conducted by the state, such as the Global Adult Tobacco Survey (GATS) and the National Family Health Survey (NFHS), which showed the alarming rates of the use of SLT in the region.

Maharashtra is one of the largest states in India with a large population and a huge regional variation with respect to the use of SLT. The rural areas were chosen for the research because the use of SLT is more common in the rural areas than the urban areas due to the deep-rooted cultural acceptance of the habit, the availability of products such as khaini, gutkha, and mishri, and the lack of awareness among the people about the harmful effects of the product.

The field research was aided by the local health centers and the gram panchayats and the health workers at the local level.

### 2.3 Study population

The target population consisted of individuals belonging to the age group of 18 to 70 years and living in the rural areas of Maharashtra state. These individuals were either current users (regardless of frequency or length of use, current users are those who had used any type of smokeless tobacco at least once in the previous 30 days) or had a prior experience of using smokeless tobacco products.

The rationale for selecting the target population was based on the epidemiological evidence showing that the initiation of use of smokeless tobacco products generally occurs during early adolescence, specially school going or young adulthood, and continues throughout life.

The demographic characteristics of the target population included individuals belonging to different age groups, genders, different socio-economic conditions, and varying levels of education.

### 2.4 Sample size and sampling technique

The study made use of a sample size of 60 participants, which was arrived at after taking into consideration the feasibility of the study and the need for in-depth analysis rather than a broad-based generalization. Although the sample size was relatively small, it was carefully stratified to ensure representativeness.

The sampling frame included residents from chosen rural areas of Maharashtra. The districts included in the study were Wardha, Chandrapur, and Yavatmal. Participants who met the study's inclusion criteria were selected in the study as study population. Participants were identified through household visits and community screening in the selected villages.

The type of sampling used in this study was stratified purposive sampling. The participants were stratified according to the following criteria:

- Age: 18–30 years, 31–50 years, and above 50 years.
- Gender: male and female.
- Socio-economic status (SES): this was measured using the modified Kuppuswamy scale, which was modified to suit the rural environment, taking into consideration education, occupation, and income.

### 2.5 Inclusion criteria

To make sure that the results are relevant and reliable, the following criteria were used for inclusion:

1. Permanent residents: participants who have been residing in the rural area of choice for at least 1 year to ensure that they are well familiarized with the community and the usage patterns of SLT.
2. Non-users of smokeless tobacco: participants who don't have any experience of SLT.
3. SLT experience: participants who have experience with smokeless tobacco, either in the form of current users (daily or occasional) or past users.
4. Age group: 18 years and above.
5. Willingness to participate: participants who are cooperative and willing to participate in the structured interviews and questionnaires.

### 2.6 Exclusion criteria

In order to ensure the relevance of the study, the following criteria were used for exclusion:

1. Serious health conditions: persons with serious health conditions (such as cancer, mental health problems, and intellectual impairments) that may impair the capacity to understand and respond to questions were excluded.
2. Unwilling participants: persons who refused to participate in the study or withdrew consent at any point during the study.

## 3 Data collection tools and procedure

The Data collection was done using a structured questionnaire which was developed from the LSAI tool and modified according to the specific socio-cultural context of rural Maharashtra. The questionnaire was pre-tested and translated into Marathi, the local language, to ensure ease of understanding and accurate responses. The content validity of the study questionnaire was assessed by a panel of subject experts from the field of Community health and related disciplines to ensure relevance, comprehensiveness and clarity of the questions. All the necessary modifications were done in the questionnaire as per expert advise.

Key sections of the questionnaire included:

- Demographic profile (age, gender, education, occupation, income).
- History of SLT use (age of initiation, frequency, duration).
- Reasons for use (peer pressure, habit, stress relief, cultural factors).
- Awareness of health consequences.
- Attempts to quit and barriers to cessation.

Interviews were conducted face-to-face by trained field investigators, with an average duration of 25–30 min per participant. Privacy and confidentiality were maintained throughout.

### 3.1 Observation and results

A total of 60 rural participants were considered for the study, and the proportion of male (50%) and female (50%) participants was equal.

### 3.2 Prevalence of smokeless tobacco use

Among the 60 participants, 40 participants (66.7%) were using smokeless tobacco, and 20 participants (33.3%) were non-users of smokeless tobacco. This clearly implies that the rural population has a high burden of smokeless tobacco use, and nearly two-thirds of the rural population was actively engaged in the use of tobacco.

Prevalence of the use of smokeless tobacco among the rural population was consistent with the data obtained from the GATS-2 survey, which reported a high use of smokeless tobacco among the rural population, i.e., 21.5%.

### 3.3 SLT use by gender

SLT use was equally distributed between males and females, with 20 users each. No statistically significant association was found between gender and SLT consumption (χ^2^ = 0.00, *p* = 1.00). This finding challenges the traditional perception of male predominance in tobacco use and suggests increasing acceptance and normalization of SLT consumption among females in rural settings (Figure 1).

**Figure 1:**
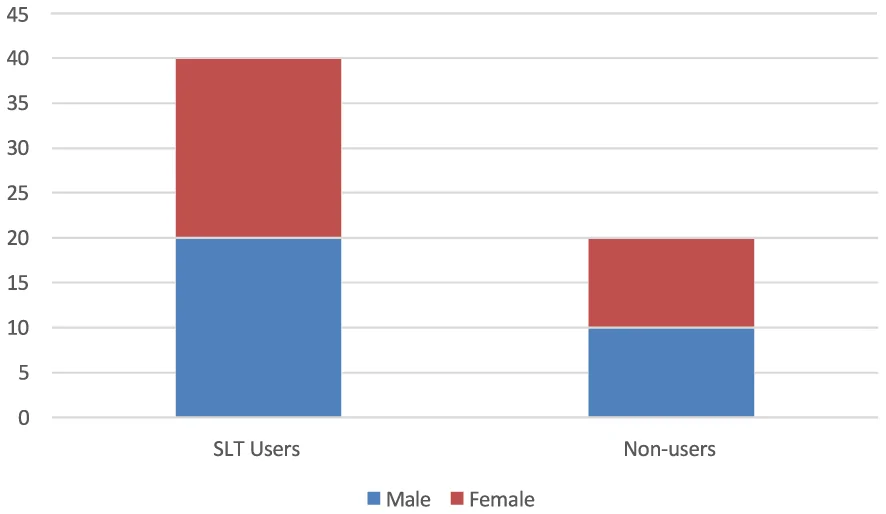
The overall study population and participant distribution.

### 3.4 SLT use by age group

The usage of SLT was found to vary across age groups. The highest prevalence was found among the 25- to 34-year-old population (21.7%). This was followed by the 45- to 54-year-old population (16.7%) and the ≥55 years age group (16.7%). A lower prevalence was found among the population below 18 years (13.3%).

A statistically significant association was found between age and the usage of SLT (χ^2^ ≈ 9.21, *p* < 0.05). Adults between 25 and 54 years accounted for over half of the population. This implies that mid-to-late adulthood is a critical period for risk Figure 2.

**Figure 2:**
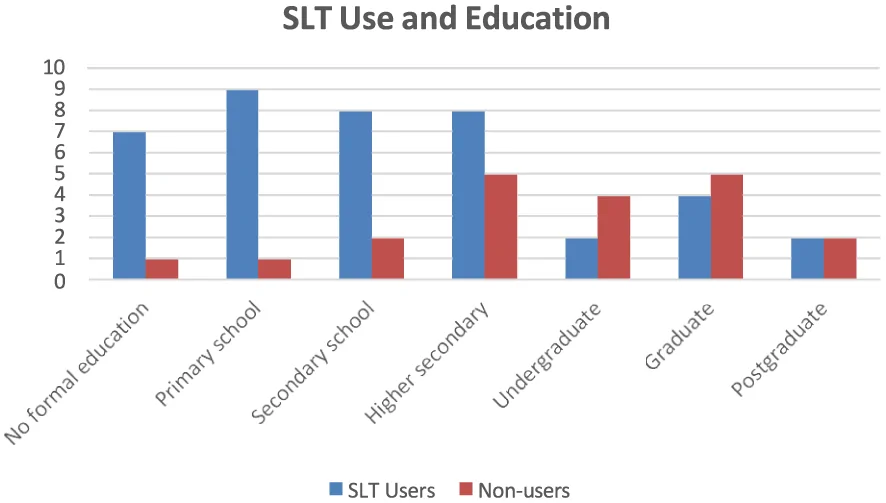
The distribution of smokeless tobacco products used by the participants.

### 3.5 SLT use by educational status

The educational attainment of the participants revealed a significant inverse relationship with the consumption of SLT. Among the participants, most of the consumers of SLT were educated to a primary level (*n* = 9) or secondary level (*n* = 8). In contrast, seven participants were not formally educated. In comparison, the non-users of SLT were more likely to be educated to a higher level.

The chi-square test revealed that there is a highly significant association between education level and SLT consumption. In this context, χ^2^ ≈ 11.32, *p* < 0.01 Figure 3.

**Figure 3:**
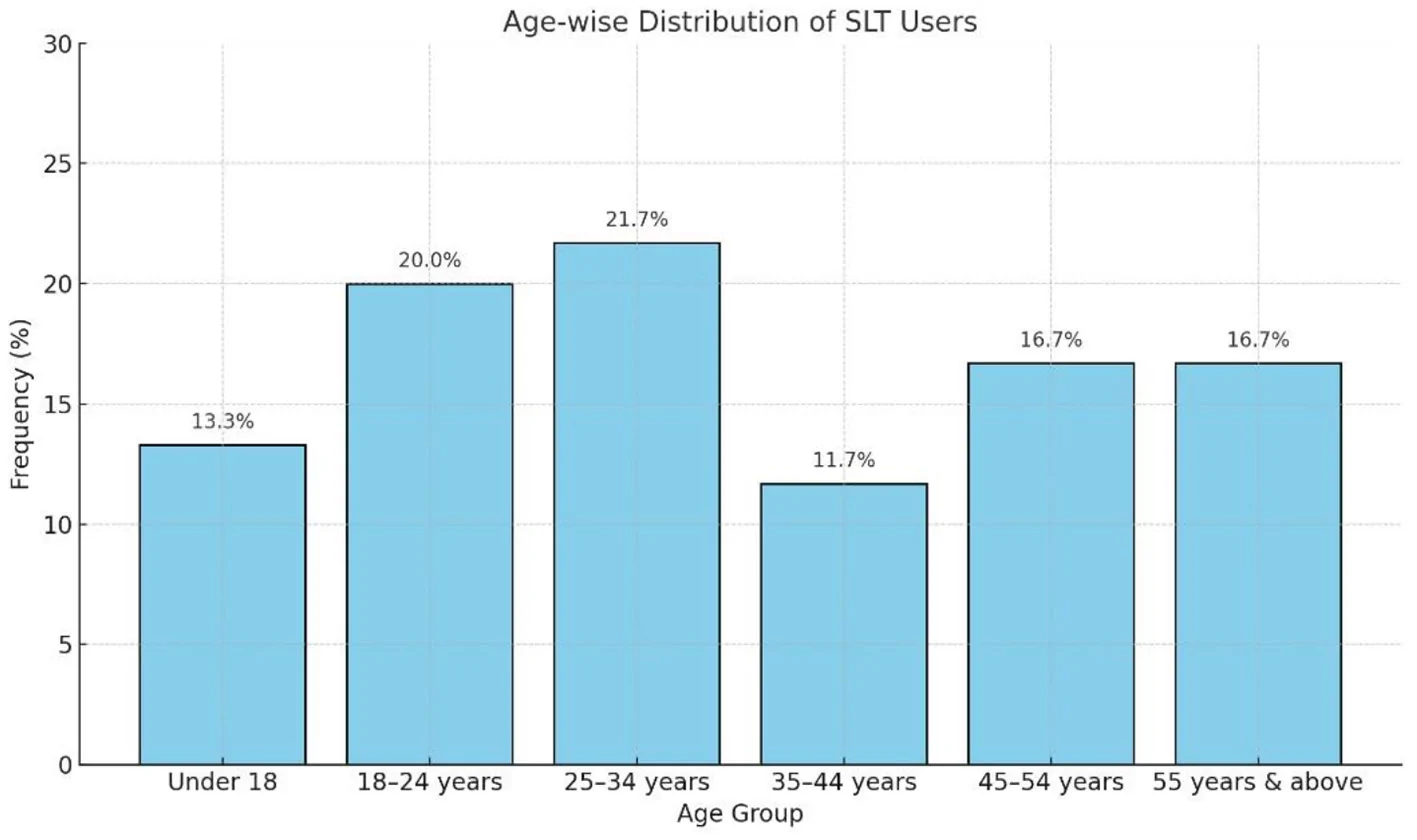
The pattern of smokeless tobacco use among the participants.

### 3.6 Occupation and SLT use

Occupation showed a significant association with SLT use (χ^2^ ≈ 8.71, *p* < 0.05). Higher usage was observed among unemployed individuals (*n* = 10), full-time workers (*n* = 10), and part-time workers (*n* = 7). Students and retired individuals demonstrated comparatively lower prevalence.

These findings suggest that employment-related stress, workplace culture, and economic pressures may contribute to tobacco use, particularly among labor-intensive and unstable occupational groups Figure 4.

**Figure 4:**
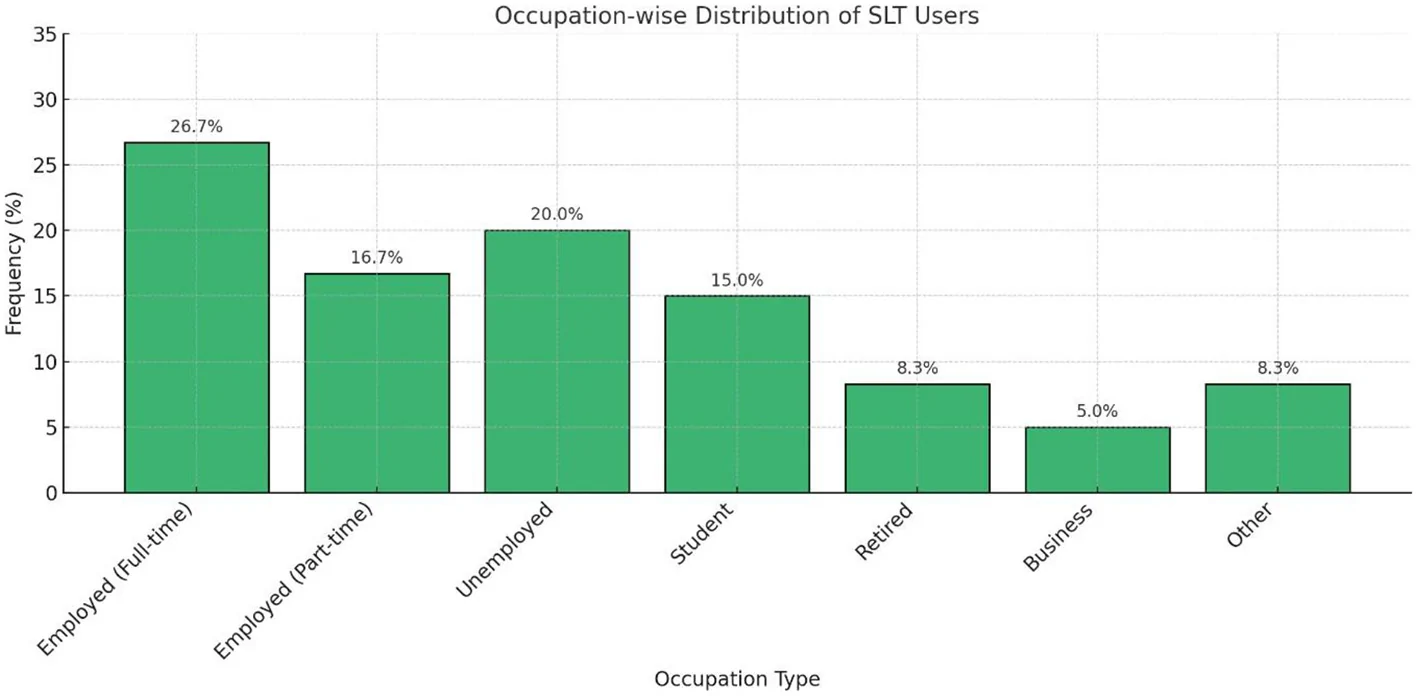
The association between socio-demographic characteristics and SLT use.

### 3.7 Income and SLT use

Monthly household income also demonstrated a significant association with SLT use (χ^2^ ≈ 7.85, *p* < 0.05). A majority of users belonged to lower income groups, particularly those earning less than 20,000 per month. Individuals in higher income categories (> 30,000) showed comparatively lower usage.

This trend indicates that lower socio-economic status may increase vulnerability to tobacco consumption due to stress, affordability of SLT products, and limited access to cessation resources Figure 5.

**Figure 5:**
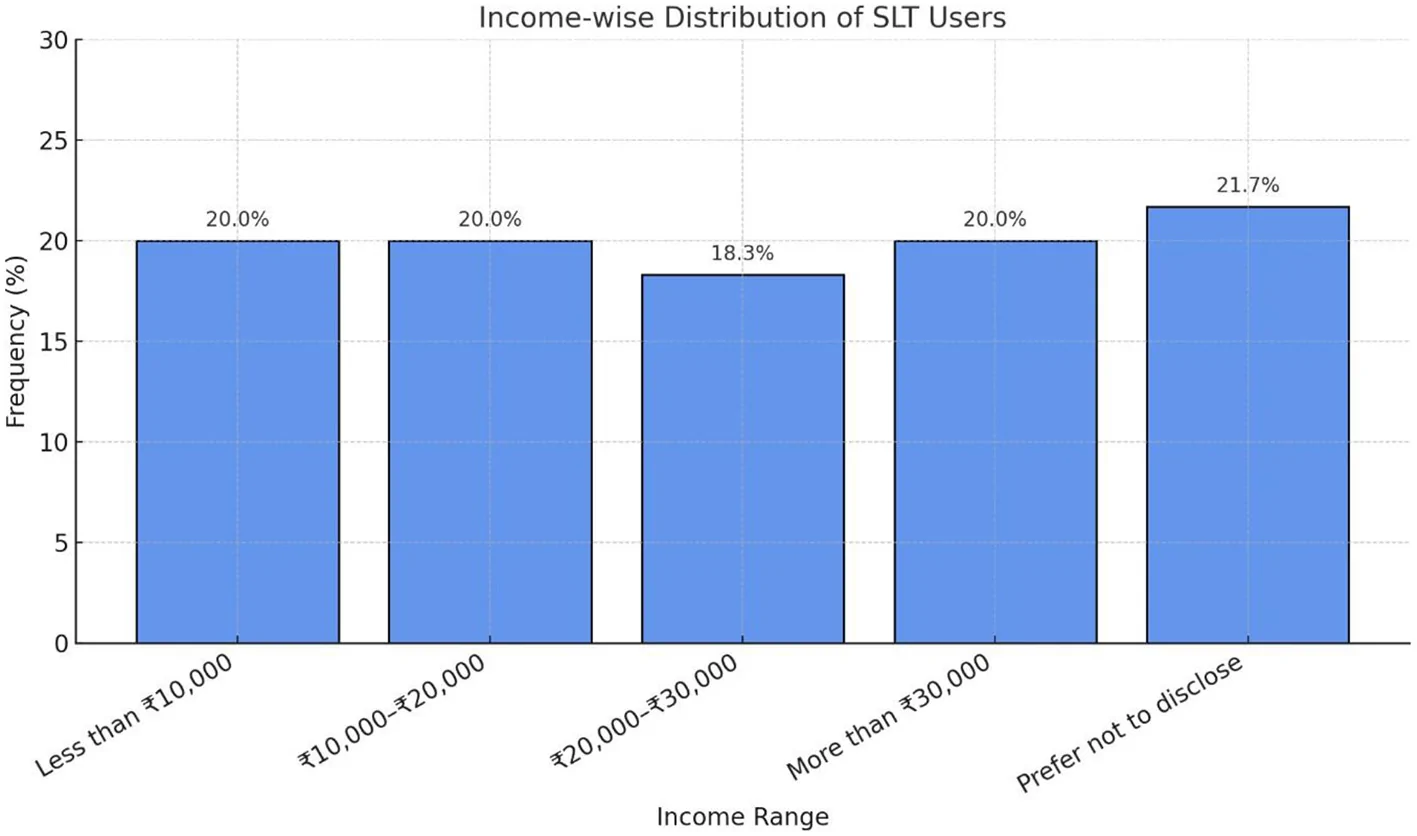
The reported health effects and/or awareness regarding SLT use.

### 3.8 Motivation for SLT use

The most common reason for the use of SLT was found to be habitual use. This was followed by stress reduction and peer pressure. Social interactions and environmental influences were also found to play a role in the initiation and maintenance of the use of SLT.

### 3.9 Health risk awareness

It was found that despite the high prevalence of SLT use, 90% of the population was aware of the health risks associated with tobacco use. These health risks include oral cancer, gum disease, and addiction. However, awareness was found to have no significant effect on the behavior of the population regarding the use of tobacco (χ^2^ = 0.00, *p* > 0.05) Figure 6. This is known as the knowledge-behavior gap.

**Figure 6:**
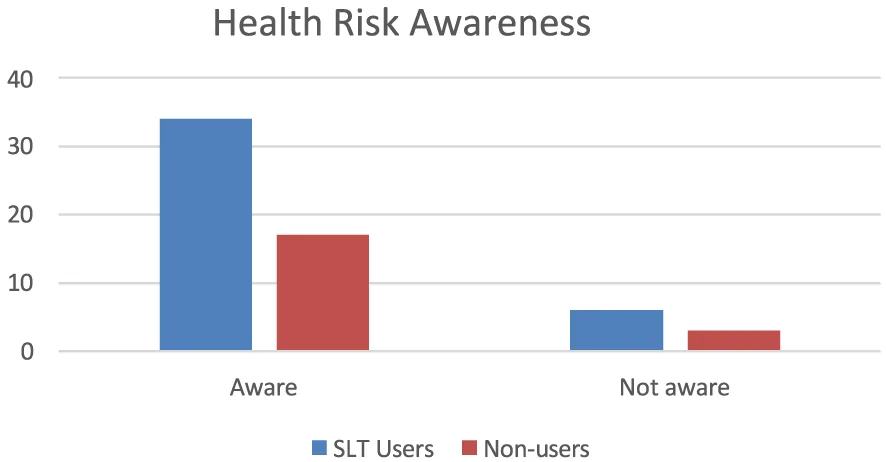
The factors influencing smokeless tobacco consumption.

### 3.10 Summary of statistical associations

Statistical analysis showed significant associations with the use of SLT and age, education, occupation, and income levels (*p* < 0.05). However, no significant association was seen with gender and awareness of health risks. Lower education levels, income levels, and unstable employment were identified as the main factors associated with the use of ST.

Thus, the results showed that the use of ST is highly prevalent among the rural adult population and is associated with socio-economic and behavioral factors rather than awareness as shown in Table 1.

**Table 1:** Summary of statistical findings.

Variable	Statistical test	χ^2^ Value	*p*-value	Significance
Gender	Chi-square test	0.00	1.00	Not significant
Age group	Chi-square test	~9.21	< 0.05	Significant
Education	Chi-square test	~11.32	< 0.01	Highly significant
Occupation	Chi-square test	~8.71	< 0.05	Significant
Income	Chi-square test	~7.85	< 0.05	Significant
Health awareness	Chi-square test	0.00	>0.05	Not significant

## 4 Discussion

The results obtained through the present research gave a significant level of insights into the patterns and determinants of the usage of smokeless tobacco among the rural populace of Central India. The results obtained through the present research are consistent with the overall body of research done so far on the topic. The discussion above gave a background on the context developed through the previous research done at the national and international level.

The research also affirms the fact that the usage of SLT is still considered a socially acceptable phenomenon among the rural populace. This is also supported by the research done by Siddiqui and Ogbeide (6), which emphasized the discreet usage of SLT among rural women. Goyal et al. (12) carried out a study on variations in the patterns of tobacco usage among Indian females—findings from the global adult tobacco survey India. This is also supported by the results obtained through the present and the study concluded that A high proportion of women consume tobacco which is significantly influenced by socio-demographic factors.

Research also which showed a significant level of female respondents who confessed to the usage of SLT for medicinal or traditional purposes, such as the relief of toothache or morning sickness, as emphasized by Saxena and Sharma (27). Halder et al. (13) reported that SLT consumption was higher among middle-aged and olderly Indian adults (15).

Most of the participants were either not literate or had limited educational qualifications, which is directly associated with the increased rate of SLT consumption. This is in line with the research conducted by (21) and Yadav and Singh (14), which showed that the less educated a person is, the higher the consumption of tobacco products. Illiteracy restricts people from accessing health information and understanding the health messages on tobacco products or anti-tobacco advertisements, as also discussed in Goel et al. (25) regarding the lack of implementation of tobacco laws.

Moreover, the results of the present study have found early initiation of SLT use, even before the age of 18 years, usually due to the influence of family and friends. Similar findings have been highlighted in the study done by (23) in which early initiation of SLT was found to start at the age of 12 years among tribal populations. Bali et al. have also highlighted the role of peer and family influence in SLT use among adolescents^1^.

In the present study, the prevalence of smokeless tobacco (SLT) use was observed to be higher in employed individuals. Such findings are consistent with the data available from the Global Adult Tobacco Survey (GATS) India, wherein it had been shown that the rate of SLT use was fairly high in employed individuals, especially those who were involved in labor-intensive occupations. Factors like occupational stress, social pressure at the workplace, low cost of SLT products, and the stimulating effects of tobacco in overcoming tiredness associated with working for prolonged periods could have contributed to this prevalence.

Respondents who were engaged in labor-intensive occupations such as farming or construction were more likely to use SLT, citing reasons such as relieving stress, suppressing hunger, or enhancing stamina. This resonates with the studies by (17) and Bhattacharjee and Ray (2019), which indicated that occupational factors and work-related stress are key contributors to the use of SLT. Furthermore, (22) indicated that widowhood, unemployment, and informal labor sectors contributed to higher levels of nicotine dependence, which resonates with the current study's findings.

Majority of the users in this study demonstrated symptoms of Nicotine Dependence and oral health problems like gum bleeding, ulcers, and difficulty in chewing. These results have been found to support the findings of other studies done by Chaturvedi et al. (24) and (20) regarding the association of SLT with an increase in oral cancer and other pre-malignant lesions. (16) found that around 40% of women in rural Odisha showed strong Nicotine Dependence.

Some participants reported SLT as a tool for mitigating psychological stress, especially among low-income male workers. This pattern was consistent with the results obtained by (18), who established the relationship between SLT use and poor mental health outcomes among rural adolescents. In fact, Kamal Sonavane et al. established the link between climate change-related stressors and substance use, providing a unique perspective on SLT dependency as a coping mechanism.

Even though a few participants have shown their willingness to quit smoking, the majority have not made any attempt to quit smoking due to lack of access to resources to quit smoking. According to Kumar et al. (28), the ineffectiveness of digital resources in helping smokers quit smoking has also been a major challenge in the country due to lack of digital literacy in the country. (19) have also suggested an integrated approach to quit smoking.

The study found that there was very little evidence of the implementation of tobacco control measures like COTPA in the areas surveyed. SLT products were easily available in the areas around schools and public spaces, similar to the findings of Goel et al. (2014)(25).

Tobacco use was found to be more prevalent in rural than urban areas and among the poorer wealth quintiles. Differences in the prevalence of various socio-economic and cultural factors were found to contribute to the rural-urban differences in tobacco consumption, as reported by Halder et al. (13).

Another major aspect of tobacco control highlighted in the literature, particularly in the article by Thankappan et al. (29), was the effectiveness of awareness campaigns (26). This aspect was also highlighted in the present study, and it was found that community influencers, ASHAs, and teachers should be utilized for tobacco control awareness campaigns. Behavioral communication may prove to be effective in tobacco control.

## 5 Conclusion

- The prevalence of SLT use among the rural population was found to be high, and the pattern of SLT use varied according to region, age group, and gender. Daily and long-term use of SLT was found, reflecting dependent rather than occasional use.
- Moreover, the use of SLT was found to be much higher among males, people with lower levels of education, and people living in poor-income families. In addition, SLT use was found to start at the teenage stage and continue through early adulthood and middle age. In contrast, the use of SLT among women was found to be underreported but was still prevalent in some areas.
- The commonly used products of SLT were khaini, gutkha, zarda, and betel quid, which varied depending upon the place and availability of the product. People used different types of SLT, unaware of the variation in the content of nicotine and risks associated with it.
- The main reasons cited for the initiation of SLT use were peer influence, family influence, curiosity, and cultural normalcy. For the continuation of the use of SLT products, addiction, stress relief, misconception about the health benefits of the product, and habit were the main factors.
- Majority of the users of SLT products were not aware of the health hazards associated with the product. Most believed that SLT was a safer option than smoking. The awareness campaigns and the health warnings on the product were not effective in the rural areas.

## Data Availability

The raw data supporting the conclusions of this article will be made available by the authors, without undue reservation.

## Footnotes

1 Shivaranjan Chandrappa Bali, Prashanth Peethala and G. Gopalakrishnan and specifically reports that peer pressure was a significant predictor of nicotine use among adolescents.
